# Socioeconomic differences in influenza vaccination coverage by government financial support status: A population-based study in Korea

**DOI:** 10.1371/journal.pone.0353764

**Published:** 2026-08-13

**Authors:** Sunhee Park, Jeoung A. Kwon, Hyo-Seon Kim, YoonJoo Choi, Byungmi Kim, Euichul Shin

**Affiliations:** 1 Department of Public Health, College of Medicine, The Catholic University of Korea, Seoul, Republic of Korea; 2 Division of Cancer Prevention, National Cancer Control Institute, National Cancer Center, Goyang, Republic of Korea; 3 Department of Preventive Medicine, Daegu Catholic University School of Medicine, Daegu, Republic of Korea; 4 Department of Preventive Medicine, College of Medicine, The Catholic University of Korea, Seoul, Republic of Korea; Yonsei University Medical Center: Yonsei University Health System, KOREA, REPUBLIC OF

## Abstract

**Background:**

Influenza can be prevented through vaccination, however vaccination coverage remains low. This study aims to analyze whether government financial support policies associated with improved vaccination coverage and to further analyze other influencing socioeconomic factors.

**Methods:**

This study utilized secondary data from the 2020 Korea National Health and Nutrition Examination Survey to analyze factors influencing influenza vaccination among 5,592 participants. Multivariate logistic regression analysis was used to analyze the association between government financial support and vaccination coverage, and to analyze socioeconomic factors influencing vaccination in both the government-supported and non-supported groups.

**Results:**

The vaccination rate was higher in the financial-supported group(63.8%) than in the non-supported group(33.1%). After adjustment, the financial assistance group had a 2.02 higher vaccination coverage compared to the non-supported group. In the financial-supported group, low education and economic inactivity were associated with higher vaccination rates. While, in the non-supported group, women, higher household-income, and having employee-insured subscribers were associated with vaccination coverage.

**Conclusions:**

Financial support is provided to high-risk groups for influenza infection, and this group showed higher vaccination coverage in low socioeconomic groups. Therefore, targeted government intervention appears necessary to improve vaccination coverage in high-risk groups.

## 1. Introduction

Seasonal influenza is an acute respiratory infection caused by influenza viruses type A or B, commonly known as the ‘flu’ [1]. This flu causes 290,000–650,000 respiratory deaths annually in the world [2], 28,000–111,500 deaths in children younger than 5 years attributable to influenza-associated acute lower respiratory infections in 2008 [3]. For healthy people, it may be considered just a severe cold, but for the elderly and those with chronic diseases, it is a dangerous disease that can even lead to death. In Korea, seasonal influenza or pneumonia mortality was 4.2%, and respiratory or circulatory mortality was 30.5% from the 2009–2010 season to the 2015–2016 season [4]. In addition, the total socioeconomic cost of seasonal influenza for the Korean adult population was estimated to be $125 million($1 = 1,100 won) during the 2013–2014 season [5].

Vaccination is the best prevention from influenza infection, especially for high-risk groups. WHO recommends the below priority groups for seasonal influenza vaccination to protect from the infection; Health workers, Older adults, Pregnant women, Individuals with specific chronic medical conditions [6,7]. Since the COVID-19 pandemic era, vaccination for herd immunity has been emphasized [8] and increased awareness of personal protection against the virus has led to increased influenza vaccination coverage [9]. Many countries are implementing government-level policies, such as policies to improve accessibility or financial support, to increase influenza vaccination coverage. In the United Kingdom, the influenza vaccine is provided free of charge by the National Health Service(NHS) for aged over 65, 2–11 years, pregnant, disabled and weakened immune system [10]. And France [11], Australia [12], Canada [13], and Japan [14] governments also provide the free vaccination for those older adults and people with severe disease.

Korea has implemented a National Immunization Program(NIP) to prevent infection from the influenza since 1997. As part of the NIP, the government has been implementing accessibility policies to increase the influenza vaccination coverage. They expanded the places from public health centers to private medical institutions, allowing many citizens to receive free vaccinations at more diverse medical institutions in 2015. However, according to previous research, the above accessibility policy did not show significant effects [15,16]. This is because while vaccination coverage increased in private hospitals, the benefits were offset by decreased vaccination coverage in public facilities [16].

Another policy to increase the influenza vaccination coverage is the financial support policy. The Korea Disease Control and Prevention Agency(KDCA) has defined the following priority influenza vaccination recommendations to prevent infection [17]: seniors over 65s, children aged 6 months to 12 years, pregnant, and those with chronic medical conditions. These priority groups have changed slightly each year, and the government has expanded these recommendations during the COVID-19 pandemic. Also, some local governments provide partial free vaccinations to medical workers, people with disabilities, and those with national merit, but this varies depending on each local government’s policies.

As a result of these policy support, South Korea had highest influenza vaccination coverage among elderly people aged 65 years and older(80.7%) among Organization for Economic Cooperation and Development(OECD) countries in 2020 [18]. Despite of high score of vaccination coverage among the elderly, the Korean Community Health Survey found that only 45.9% of the overall population received vaccinated in 2020 [19], indicating that more than half of the population remained unvaccinated.

Socioeconomic status(SES) disparity plays a significant role in preventive behaviors such as vaccination. Education level influences health-related decision-making. Knowledge of the effectiveness of appropriate vaccines often leads to vaccination behavior, income and economic activity influence preventive care such as purchasing healthy foods, gym memberships, and vaccinations [20]. Research supported this finding, showing that higher education and income levels are associated with higher vaccination coverage [21,22].

Previous studies have shown that government financial support policies are the most important factor in increasing vaccination coverage [15,16]. However, given the government’s limited health budget [5,23], it is difficult to indefinitely expand financial support policies, and an understanding the various socioeconomic factors that influence vaccination is also necessary. One study analyzed socioeconomic factors influencing influenza vaccination trends and behavioral decisions [24], and another study identified socioeconomic factors influencing influenza vaccination during the COVID-19 pandemic, but these studies did not examine the impact of government financial support [21].

Therefore, this study aims to analyze whether government financial support policies are associated with increased vaccination coverage and to identify socioeconomic factors associated with vaccination coverage between government-supported and non-supported groups [25].

## 2. Materials and methods

### 2.1 Data source and study population

This study was based on data collected by the Korean National Health and Nutrition Examination Survey(KNHANES) in 2020. The KNHANES is a cross-sectional survey conducted since 1998 by the Korea Disease Control and Prevention Agency and the Ministry of Health and Welfare. KNHANES uses a complex, multistage probability sample design, and sample weights were applied in all analyses to produce estimates representative of the Korean population [26,27]. Trained interviewers administered questionnaires on various health-related information, and respondents self-reported their health and vaccination status in face-to-face interviews.

**Fig 1:**
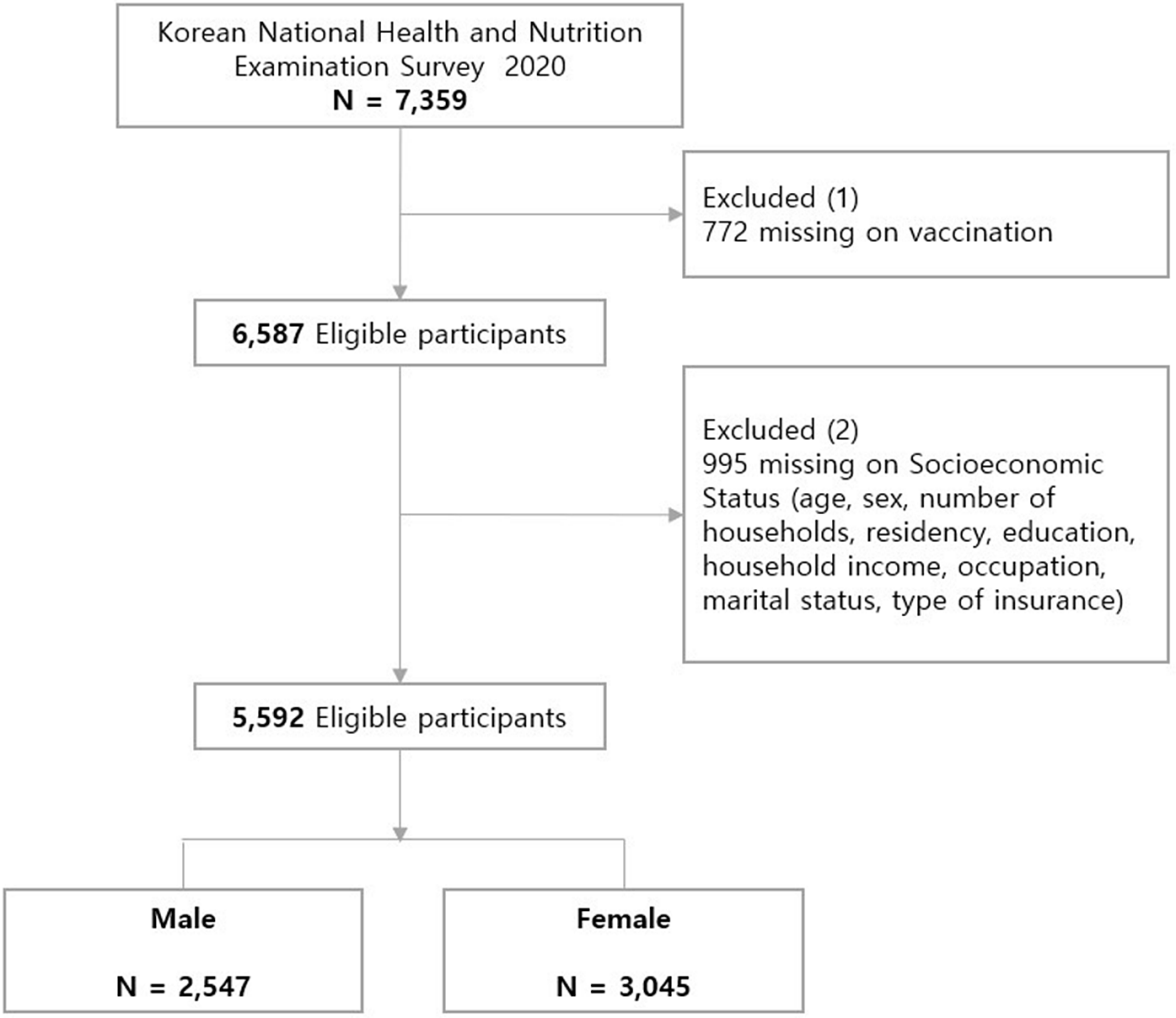
Flow chart of study population.

Among the 7,359 subjects that participated in KNHANES, the exclusion criteria were as follows: 1) participants with missing vaccination information(n = 772); 2) participants with missing socioeconomic status information(age, sex, residency area, level of education, monthly household income, occupation, marital status, number of households, type of health insurance)(n = 995). In this study, we included 5,592 participants(2,547 of Men, 3,045 of Women) (Fig 1 and S1 Table).

### 2.2 Study design

Based on the NIP, we designed and analyzed the study as follows:

1) Assess the characteristics of the vaccinated group and the unvaccinated group.
2) Investigate the factors associated with influenza vaccination, such as financial support or not, and socioeconomic status, a multivariable logistic regression was performed.
3) For subgroup analysis, we divided the entire population into two groups: financial support and non-financial support. Multivariable logistic regression was used for each group analysis, and the factors associated with each socioeconomic status were identified.

### 2.3 Outcome variable

The outcome variable was seasonal influenza vaccination status. The KNHANES asked participants about vaccination: “Have you been vaccinated against influenza(seasonal flu) in the past year?” Respondents could answer “yes” or “no” in dichotomous (S2 Table).

### 2.4 Financial support group

The primary exposure of this study is government financial support for influenza vaccination. Generally, the Korean government recommends vaccination through financial support for children(under 12 years of age), pregnant women, the elderly(over 65 years of age). And individuals with chronic diseases, such as patients with lung, kidney, neuromuscular, oncological, heart disease, diabetes, and immunocompromised, individuals receiving treatment in group facilities due to chronic disease were recommended because they were at high-risk for influenza virus infection. However, during the 2020−2021 COVID-19 pandemic, the age criteria were temporarily expanded for the following reasons: 1) To prevent influenza outbreaks caused by communal living, the existing age criterion for children aged 6 months to 59 months was expanded to include children and adolescents aged 6 months to 18 years. 2) To prevent community influenza outbreaks, the existing age criterion for seniors aged 65 and older was expanded to include seniors aged 62 and older [17].

Therefore, this study classified the following individuals as eligible for vaccination if they received free government-sponsored vaccination during the 2020–2021 season (S3 Table).;

1) Age criteria: Children and adolescents under 18 years of age, and the elderly over 62 years of age.
2) Pregnant women: 10 of the study participants were identified as pregnant, and all were considered to have received free government-sponsored vaccination.
3) Individuals with chronic diseases: Following government recommendations, individuals diagnosed with diabetes, heart disease(angina pectoris, myocardial infarction, and stroke), lung disease(pulmonary tuberculosis and asthma), liver disease(hepatitis B, hepatitis C, and cirrhosis), kidney disease, and cancer(stomach, liver, colon, breast, cervical, lung, and others excluding thyroid) were classified as having chronic diseases [17].

### 2.5 Socioeconomic status

We considered the following covariates: sex, number of households, residency area, education level, monthly household income, economic activity, marital status, type of health insurance. The number of household members were classified as “1 person” if there was only one; if the number of members was over 2, classified as “2+ persons”. Residency area was classified as “Urban(‘Dong’ Unit)” and “Rural(‘Myeon’, ‘Eup’ Unit)”. The educational level was classified into three levels; as less than middle school graduate, high school graduate, and college(university) graduate or higher. The monthly household income classified into quartiles(quintiles 1, 2, 3, and 4). The economic activities were classified as “yes” or “no”. The type of health insurance was classified into three groups; employee-insured, self-employed-insured, and medical aid. South Korea operates a universal, single-payer National Health Insurance(NHI) system that covers the entire population. Employees contribute jointly with employers, the self-employed pay household-based contributions, and medical aid beneficiaries receive government-funded support.

### 2.6 Statistical analysis

SAS software version 9.4 (SAS Institute, Cary, NC, USA) was used for all data analyses. For categorical variables, the chi-square test was used to compare the proportions of sociodemographic characteristics between vaccinated and unvaccinated individuals. Multivariate logistic regression was used to analyze the impact on vaccination rates after adjusting for covariates (Proc Surveylogistic). Data from the Korea National Health and Nutrition Examination Survey were extracted using a two-stage stratified cluster sample design, and strata(Kstrata), clusters(PSU), and weights(wt_itvex) were considered and reflected in the analysis.

### 2.7 Ethical statement

All participants provided written informed consent before participating in the Korean National Health and Nutrition Examination Survey(KNHANES), which was approved by the Institutional Review Board of the Korea Disease Control and Prevention Agency(IRB No. 2018-01-03-2C-A).

## 3. Results

### 3.1 Study and participant characteristics

Among the 5,592 participants, 47.0% were vaccinated, while 53.0% were unvaccinated(Tables 1 and S1). In financial support group, 63.8% were vaccinated, while, in non-support group, 33.1% were vaccinated. Among the study participants, women(50.7%), those living alone(53.5%), urban residents(49.5%), those with an education level of less than middle school graduation(67.1%), those in the lowest income quintile(60.9%), the economically inactive population(53.8%), elementary occupations(57.9%), married people(53.3%), those diagnosed with chronic diseases(61.4%), medical aid(57.2%), and pregnant women(50.0%) showed relatively high influenza vaccination coverage.

**Table 1:** Demographic and socioeconomic characteristics by vaccination status.

	Total	Vaccinated		Unvaccinated		P-value
		N (%)		N (%)		
Total	5,592	2,626	(47.0)	2,966	(53.0)	
Financial Support Status						
Financial Support group †	2,520	1,608	(63.8)	912	(36.2)	<.0001
Non-support group	3,072	1,018	(33.1)	2,054	(66.9)	
Sex						
Male	2,547	1,081	(42.4)	1,466	(57.6)	<.0001
Female	3,045	1,545	(50.7)	1,500	(49.3)	
Number of Households Member						
1Person	703	376	(53.5)	327	(46.5)	0.000
2 + Persons	4,889	2,250	(46.0)	2,639	(54.0)	
Residency area						
Urban	1,097	543	(49.5)	554	(50.5)	0.060
Rural	4,495	2,083	(46.3)	2,412	(53.7)	
Level of Education						
Less than middle school	1,616	1,085	(67.1)	531	(32.9)	<.0001
High school	1,927	780	(40.5)	1,147	(59.5)	
College or more	2,049	761	(37.1)	1,288	(62.9)	
Household income						
1st (lowest) quartile	944	575	(60.9)	369	(39.1)	<.0001
2nd quartile	1,333	690	(51.8)	643	(48.2)	
3rd quartile	1,587	675	(42.5)	912	(57.5)	
4th (highest) quartile	1,728	686	(39.7)	1,042	(60.3)	
Economic activity status						<.0001
Yes (Employed)	3,259	1,370	(42.0)	1,889	(58.0)	
No (Unemployed)	2,333	1,256	(53.8)	1,077	(46.2)	
Marital status						<.0001
Married	4,254	2,266	(53.3)	1,988	(46.7)	
Unmarried	1,338	360	(26.9)	978	(73.1)	
Health insurance type						
Employee-Insured	1,680	750	(44.6)	930	(55.4)	0.001
Self-Employed-Insured	3,676	1,741	(47.4)	1,935	(52.6)	
Medical Aid	236	135	(57.2)	101	(42.8)	
Chronic disease *						<.0001
Yes (Diagnosed)	1,365	838	(61.4)	527	(38.6)	
No (Not- Diagnosed)	4,227	1,788	(42.3)	2,439	(57.7)	
Pregnancy						
No	5,582	2,621	(47.0)	2,961	(53.1)	0.847
Yes	10	5	(50.0)	5	(50.0)	

†: Financial Support Group: Individuals of Older Adults, Pregnant Women, Chronic Disease. In general, free vaccinations were provided to adults aged 65 and older, but during the 2019–2020 season when the coronavirus was prevalent, free vaccinations were temporarily expanded to adults aged 62 and older.

*Chronic Disease Diagnosed: Diabetes mellitus, Heart diseases (Coronary Heart Disease, Myocardial Infarction, Angina, Stroke), Lung Diseases (Pulmonary Tuberculosis, Bronchial Asthma, Chronic Bronchitis, Emphysema), Liver Diseases (Chronic Hepatitis, Liver Cirrhosis), Kidney Diseases (Chronic Renal Disease), Cancer (Stomach, Liver, Colon, Breast, Cervical, Lung, Other cancer exclude Thyroid Cancer).

Displaying socioeconomic factors by age, the 62-year-old age group had the highest prevalence(31.0%). The age group with the highest rate of vaccination was also 62 and older. Discovering education levels by age, those in their 30s to 49s were most likely to have a college degree or higher(51.5%), while those with a middle school diploma or lower were most likely to have been 62 and older(69.2%). In terms of the distribution of household income by age, the highest quartile was the 30–49 age group(36.3%), while the lowest quartile was the 62 + age group(66.3%). The largest economically active age group was the 30–49 age group(38.1%), while the economically inactive age group was the 62 + age group(43.7%). Pregnant women were distributed exclusively in the 19–49 age group, and chronic disease patients were most prevalent among those 62+(58.5%) and least prevalent among those under 18 years(0.7%).

### 3.2 Factors associated to influenza vaccination

Table 2 showed the adjusted OR of seasonal influenza vaccination coverage in total study participants group. Financial support group were vaccinated significantly higher than non-support group(aOR 2.02; 1.72–2.38). Women were higher vaccinated than men(aOR 1.27; 1.09–1.47). Higher education had lower vaccination coverage, it was showed dose-response patterns. The individuals who don’t work, married, and who had employee-insured were more vaccinated.

**Table 2:** Factors associated to influenza vaccination status as assessed through multivariable logistic regression.

		OR (95% CI)	
Financial support Status	Non-support group	1.00	
	Financial support group † *	2.02	(1.71 - 2.38)
Sex	Male	1.00	
	Female *	1.27	(1.09 - 1.47)
Number of Households Member	1person	1.00	
	2 + persons	0.92	(0.71 - 1.19)
Residency area	Urban	1.00	
	Rural	0.95	(0.76 −1.19)
Level of Education	Less than middle school	1.00	
	High school *	0.63	(0.51 - 0.77)
	College or more *	0.57	(0.47 - 0.70)
Household Income	1st (lowest) quartile	1.00	
	2nd quartile	1.10	(0.85 - 1.43)
	3rd quartile	1.00	(0.77 - 1.29)
	4th (highest) quartile	1.07	(0.81 - 1.41)
Economic Activity Status	Yes (Employed)	1.00	
	No (Unemployed) *	1.26	(1.08 - 1.46)
Marital Status	Married	1.00	
	Unmarried *	0.43	(0.35 - 0.51)
Health Insurance Type	Employee-Insured	1.00	
	Self-Employed-Insured *	0.75	(0.63 - 0.89)
	Medical Aid	0.83	(0.61 - 1.13)

†: Financial Support Group: Individuals of Older Adults, Pregnant Women, Chronic Disease. In general, free vaccinations were provided to adults aged 65 and older, but during the 2019–2020 season when the coronavirus was prevalent, free vaccinations were temporarily expanded to adults aged 62 and older.

*: Statistically significant.

### 3.3 Difference between the financially supported group and the non-funded group

For subgroup analysis, we divided the participants into two groups: the financially supported group and the non-financially supported group. In addition, we analyzed crude ratio in model 1 and adjusted odd ratio in model 2 to assess the factors associated with influenza vaccination coverage.

Table 3 shows the associations between influenza vaccination status and sociodemographic factors that influenced influenza vaccination among participants who received government funding. Adjusted odds ratios showed that higher education levels were associated with lower influenza vaccination coverage, while economically inactive individuals and married individuals had higher vaccination coverage.

**Table 3:** Factors associated to influenza vaccination status as assessed through multivariable logistic regression in financial support group (free vaccination).

		Model 1		Model 2	
		OR (95% CI)		aOR (95% CI)	
Sex	Male	1.00		1.00	
	Female	1.53	(1.25 - 1.88)	1.11	(0.88 - 1.41)
Number of Households Member	1person	1.00		1.00	
	2 + persons	0.68	(0.51 - 0.91)	0.92	(0.65 - 1.29)
Residency area	Urban	1.00		1.00	
	Rural	1.12	(0.80 - 1.57)	0.86	(0.64 - 1.15)
Level of education	Less than middle school	1.00		1.00	
	High school *	0.59	(0.45 - 0.77)	0.60	(0.45 - 0.80)
	College or more *	0.36	(0.27 - 0.47)	0.35	(0.26 - 0.48)
Household income	1st (lowest) quartile	1.00		1.00	
	2nd quartile	0.76	(0.54 - 1.06)	1.01	(0.72 - 1.42)
	3rd quartile	0.48	(0.35 - 0.65)	0.76	(0.54 - 1.06)
	4th (highest) quartile	0.43	(0.31 - 0.59)	0.81	(0.55 - 1.19)
Economic activity status	Yes (Employed)	1.00		1.00	
	No (Unemployed) *	1.58	(1.32 - 1.90)	1.64	(1.31 - 2.06)
Marital Status	Married				
	Unmarried *	0.31	(0.24 - 0.41)	0.23	(0.16 - 0.32)
Health Insurance Type	Employee-Insured	1.00		1.00	
	Self-Employed-Insured	1.02	(0.83 - 1.25)	0.84	(0.67 - 1.05)
	Medical Aid	1.47	(0.96 - 2.24)	1.04	(0.66 - 1.62)

- Model1: Unadjusted/ Model2: Adjusted all Covariates.

*: Statistically significant in adjusted model.

Table 4 shows the associations among participants who did not receive government funding. Adjusted odds ratios also showed that higher vaccination coverage were observed among women, those with higher household incomes, those who were married, and those with employee-insured subscribers.

**Table 4:** Factors associated to influenza vaccination status as assessed through multivariable logistic regression in non-support group (self-paid vaccination).

		Model 1		Model 2	
		OR (95% CI)		aOR (95% CI)	
Sex	Male	1.00		1.00	
	Female *	1.43	(1.19 - 1.73)	1.32	(1.09 - 1.60)
Number of Households Member	1person	1.00		1.00	
	2 + persons	1.73	(1.18 - 2.54)	1.13	(0.75 - 1.70)
Residency area	Urban	1.00		1.00	
	Rural	0.92	(0.69 - 1.24)	0.97	(0.71 - 1.34)
Level of education	Less than middle school	1.00		1.00	
	High school	0.88	(0.64 - 1.21)	0.96	(0.69 - 1.33)
	College or more	0.98	(0.72 - 1.34)	0.96	(0.70 - 1.34)
Household income	1st (lowest) quartile	1.00		1.00	
	2nd quartile *	2.53	(1.66 - 3.87)	1.92	(1.22 - 3.03)
	3rd quartile *	2.63	(1.75 - 3.94)	1.97	(1.25 - 3.10)
	4th (highest) quartile *	2.78	(1.83 - 4.22)	2.06	(1.28 - 3.32)
Economic activity status	Yes (Employed)	1.00		1.00	
	No (Unemployed)	0.96	(0.80 - 1.16)	1.08	(0.88 - 1.32)
Marital Status	Married	1.00		1.00	
	Unmarried *	0.53	(0.42 - 0.65)	0.56	(0.45 - 0.70)
Health Insurance Type	Employee-Insured	1.00		1.00	
	Self-Employed-Insured *	0.70	(0.55 - 0.90)	0.73	(0.56 - 0.96)
	Medical Aid	0.30	(0.15 - 0.61)	0.51	(0.23 - 1.13)

- Model1: Unadjusted/ Model2: Adjust all Covariates.

*: Statistically significant in adjusted model.

Model fit was assessed using Akaike’s Information Criterion (AIC). The intercept-only model had an AIC of [20695164], whereas the fully adjusted model had a lower AIC of [20528004]. The reduction in AIC indicates improved model fit. Therefore, the fully adjusted model (Model 2) was selected for inference (S4–S6 Tables in S1 File).

## 4. Discussion

This population-based cross-sectional study examined the association between sociodemographic factors and influenza vaccination by categorizing according to government financial support using Korea National Health and Nutrition Survey 2020.

In this study, 47.0% participants were vaccinated (Table 1). While this figure represents a slight increase from previous figures [24,28], it still represents more than half of the overall population remaining unvaccinated.

The Korean government’s financial support policy to protect high-risk groups from influenza infection has been gradually expanded based on evidence since 1997 [29]. Our analysis revealed that the group receiving government financial support had 2.02 times higher vaccination coverage than the group not receiving financial support. This results were consistent with previous studies, suggesting that financial support was effective in increasing vaccination coverage [21,24].

In multivariable logistic regression on all subjects (Table 2), there were significant association in financial-support, sex, education level, economic activities, marital status, health insurance type. In the government-supported and non-supported groups, which were divided into subgroups, differences were observed in the factors influencing vaccination. In the financial support group (Table 3), there were association in education level, economic activities, marital status. In the non-financial support group (Table 4), there were association in sex, income level, marital status variables, and health insurance type variables.

In the government financial support group, the higher education levels tend to have lower vaccination. In the non-financial support group, the higher income levels tend to have significantly higher vaccination with dose-response pattern. Women had higher, employee health insurance type had higher vaccination coverage than self-employed health insurance type. In both group, unmarried group showed significantly lower vaccination coverage.

In both the overall participant and non-support group analyses, women were significantly more likely to be vaccinated than men, a finding consistent with previous studies [30,31]. The high vaccination coverage among women is particularly noteworthy, as many countries, including the WHO, classify pregnant women as a high-risk group for influenza [32].

Higher education was associated with lower vaccination coverage in the financial assistance group. Generally, higher education levels are expected to lead to positive health behaviors [33]. However, the results of this study, consistent with previous studies, showed the opposite pattern in vaccination behavior [28,34–36]. This may be explained by the fact that free vaccinations were primarily provided to older adults, and that older adults tended to have lower education levels [36]. Therefore, future research should investigate the association between education level and vaccination coverage in age groups other than the elderly.

The household income level showed a significant association in both the financially supported and non-supported groups, but in opposite directions. In the financially supported group, the pre-adjusted analysis revealed that lower income groups had higher vaccination coverage, while in the non-financially supported group, higher income groups had higher vaccination coverage. These results suggest that financial assistance policies for vaccination have been effective in low-income populations. Furthermore, in the non-financial assistance group, higher income levels were associated with higher vaccination coverage [36], suggesting a close relationship between income and vaccination [37]. Previous research has also shown that those with lower socioeconomic status are more flexible to price-based policies [38]. This suggests that price-based financial assistance policies should be targeted to lower social strata, such as low-income households.

The marital status in both groups was significantly related, the vaccination coverage decreased for single participants, similar to a previous study [37]. Married people engage in healthier behaviors than single people, suggesting that mutual support for health is beneficial.

We found an association between health insurance type and vaccination coverage. In both the overall participant analysis and the non-financial support group, self-employed participants had lower vaccination coverage than employed participants. Previous research has shown that employed participants share health information with coworkers and are influenced by their health behaviors, leading to healthier behavior [20]. This result suggests that self-employed individuals, who do not share peer and environmental influences, exhibit lower health behaviors and vaccination coverage compared to employed individuals. Furthermore, since the results for medical aid recipients in this study were insignificant, further analysis of low-income groups is warranted in the future.

The economic activity variable did not show significant results in the non-financial support group, but within the financial support group, the economically inactive population showed a higher vaccination coverage than the economically active population. This result is similar to the results for populations sensitive to price-based policies mentioned above [38]. The economically inactive population appears to have shown a tendency to engage in health behaviors when receiving financial support, while conversely, in the absence of financial support, no improved health behaviors were observed.

In summary, the group receiving government financial support included pregnant women, the elderly, and those with chronic diseases, all of whom are considered high-risk groups. Our study found that government-funded free influenza vaccinations were effective in increasing vaccination coverage in high-risk groups. An analysis of factors influencing non-vaccination among those classified as high-risk and eligible for free vaccination revealed that those with low education, the economically inactive, and married individuals had higher vaccination coverage. This finding confirms that government support was appropriately provided to high-risk groups with low social status. Furthermore, assuming that the group not receiving government support represents the general population, we found that groups representing vulnerable groups, those with limited access to healthcare, or those with poor health behaviors—males, low-income, unmarried, and those with local insurance—had lower vaccination coverage.

The ultimate goal of government support lies in increasing vaccination coverage in high-risk groups while simultaneously providing financial support to socioeconomically vulnerable groups. Consequently, we found a need to expand targeted policies that can promote healthcare access and health behaviors among vulnerable populations through government financial support.

This study also has several limitations. First, since influenza vaccination status was determined through a self-reporting method based on respondents’ recalls of vaccinations over the past 12 months, there is a possibility of recall bias. Second, because the survey was conducted over the entire year of 2020 (January to December), the 12-month recall period covers two flu seasons (2019–2020 and 2020–2021) and aligns only partially with the 2020 seasonal influenza vaccination guidelines published in July 2020. This temporal overlap may lead to undifferentiated exposure misclassification. Finally, due to the cross-sectional study design, it is not possible to infer causal relationships regarding policy effects. Third, there may be misclassification bias in classifying individuals who answered “yes” to the question about whether they had a specific chronic disease as eligible for financial assistance. Forth, individuals were not directly asked about financial support for vaccination, and classification according to national recommendations may also result in misclassification bias. Furthermore, we considered the financial support group that who received the central government’s policies, however, we couldn’t reflect the individuals who received the local government’s support.

## 5. Conclusion

This study demonstrates that government financial support is associated with higher influenza vaccination coverage. Furthermore, the effectiveness of government-supported vaccines was observed in lower socioeconomic populations, while higher vaccination coverage was observed in groups without government supports, particularly those with higher socioeconomic populations. These findings suggest the need for tailored policy interventions that specifically address low socioeconomic groups and high-risk unvaccinated populations.

## Supporting information

S1 Table Demographic and socioeconomic characteristics by age group. (DOCX)

S2 Table Questionnaire and variables of Korea National Health and Nutrition Examination Survey. (DOCX)

S3 Table Definition of the government financial support group for influenza in the 2021 Korean National Immunization Program. (DOCX)

S1 File S4–S6 Table. Supplementary analyses of model fit, multicollinearity, and interaction effects. (DOCX)
