# Supplementary material for: Socioeconomic differences in influenza vaccination coverage by government financial support status: A population-based study in Korea
Source: PLoS One. 2026 Aug 13;21(8):e0353764. doi: 10.1371/journal.pone.0353764 (PMC13472430; doi:10.1371/journal.pone.0353764)
Supplement: Supplementary file 1 [file pone.0353764.s001.docx]

Supporting Information 1. Demographic and Socioeconomic Characteristics by Age group

|  | | Total | Age | | | | | | | | | |
| --- | --- | --- | --- | --- | --- | --- | --- | --- | --- | --- | --- | --- |
|  |  |  | =<18 years | | 19-29 years | | 30-49 years | | 50-61 years | | 62 years =< | |
|  |  |  | N | (%) | N | (%) | N | (%) | N | (%) | N | (%) |
| Total |  | 5,592 | 218 | (3.9) | 770 | (13.8) | 1,638 | (29.3) | 1,231 | (22.0) | 1,735 | (31.0) |
| Vaccination | Yes (Vaccinated) | 2,626 | 93 | (3.5) | 190 | (7.2) | 596 | (22.7) | 450 | (17.1) | 1,297 | (49.4) |
|  | No (Unvaccinated) | 2,966 | 125 | (4.2) | 580 | (19.6) | 1,042 | 35.1) | 781 | (26.3) | 438 | (14.8) |
| Financial Support | Financial Support Group | 2,520 | 218 | (8.7) | 50 | (2.0) | 194 | (7.7) | 323 | (12.8) | 1,735 | (68.9) |
|  | Non-Support Group | 3,072 | - | - | 720 | (23.4) | 1,444 | (47.0) | 908 | (29.6) | - | - |
| Sex | Male | 2,547 | 129 | (5.1) | 384 | (15.1) | 713 | (28.0) | 554 | (21.8) | 767 | (30.1) |
|  | Female | 3,045 | 89 | (2.9) | 386 | (12.7) | 925 | (30.4) | 677 | (22.2) | 968 | (31.8) |
| Number of Households Member | 1Person | 703 | - | - | 74 | (10.5) | 146 | (20.8) | 113 | (16.1) | 370 | (52.6) |
|  | 2+Persons | 4,889 | 218 | (4.5) | 696 | (14.2) | 1,492 | (30.5) | 1,118 | (22.9) | 1,365 | (27.9) |
| Residency Area | Rural | 1,097 | 32 | (2.9) | 96 | (8.8) | 262 | (23.9) | 235 | (21.4) | 472 | (43.0) |
|  | Urban | 4,495 | 186 | (4.1) | 674 | (15.0) | 1,376 | (30.6) | 996 | (22.2) | 1,263 | (28.1) |
| Education Level | Less than Middle School Graduate | 1,616 | 184 | (11.4) | 4 | (0.3) | 44 | (2.7) | 266 | (16.5) | 1,118 | (69.2) |
|  | High School Graduate | 1,927 | 34 | (1.8) | 454 | (23.6) | 538 | (27.9) | 498 | (25.8) | 403 | (20.9) |
|  | College (University) Graduate or Higher | 2,049 | - | - | 312 | (15.2) | 1,056 | (51.5) | 467 | (22.8) | 214 | (10.4) |
| Household Income | 1st(lowest) quartile | 944 | 14 | (1.5) | 73 | (7.7) | 107 | (11.3) | 124 | (13.1) | 626 | (66.3) |
|  | 2^nd^ quartile | 1,333 | 68 | (5.1) | 154 | (11.6) | 366 | (27.5) | 232 | (17.4) | 513 | (38.5) |
|  | 3^rd^ quartile | 1,587 | 74 | (4.7) | 243 | (15.3) | 538 | (33.9) | 370 | (23.3) | 362 | (22.8) |
|  | 4th(highest) quartile | 1,728 | 62 | (3.6) | 300 | (17.4) | 627 | (36.3) | 505 | (29.2) | 234 | (13.5) |
| Economic Activity | Yes (Employed) | 3,259 | 22 | (0.7) | 416 | (12.8) | 1,240 | (38.1) | 866 | (26.6) | 715 | (21.9) |
|  | No (Unemployed) | 2,333 | 196 | (8.4) | 354 | (15.2) | 398 | (17.1) | 365 | (15.7) | 1,020 | (43.7) |
| Occupation | Managers, professionals, technicians and associated professionals | 768 | 1 | (0.1) | 128 | (16.7) | 406 | (52.9) | 176 | (22.9) | 57 | (7.4) |
|  | Clerks | 557 | - | - | 76 | (13.6) | 323 | (58.0) | 125 | (22.4) | 33 | (5.9) |
|  | Service/sales workers | 749 | 12 | (1.6) | 143 | (19.1) | 230 | (30.7) | 236 | (31.5) | 128 | (17.1) |
|  | Agricultural, forestry, and fishery workers | 187 | - | - | 2 | (1.1) | 10 | (5.4) | 44 | (23.5) | 131 | (70.1) |
|  | Craft workers, equipment and machine operation and assembling workers | 502 | 4 | (0.8) | 30 | (6.0) | 179 | (35.7) | 175 | (34.9) | 114 | (22.7) |
|  | elementary occupations | 496 | 5 | (1.0) | 37 | (7.5) | 92 | (18.6) | 110 | (22.2) | 252 | (50.8) |
|  | Unemployed (housewife, student, etc.) | 2,333 | 196 | (8.4) | 354 | (15.2) | 398 | (17.1) | 365 | (15.7) | 1,020 | (43.7) |
| Marital Status | Married | 4,254 | - | - | 59 | (1.4) | 1,277 | (30.0) | 1,196 | (28.1) | 1,722 | (40.5) |
|  | Unmarried | 1,338 | 218 | (16.3) | 711 | (53.1) | 361 | (27.0) | 35 | (2.6) | 13 | (1.0) |
| Type of Health Insurance | Employee-Insured | 1,680 | 46 | (2.7) | 183 | (10.9) | 441 | (26.3) | 406 | (24.2) | 604 | (36.0) |
|  | Self-Employed-Insured | 3,676 | 160 | (4.4) | 556 | (15.1) | 1,165 | (31.7) | 789 | (21.5) | 1,006 | (27.4) |
|  | Medical Aid | 236 | 12 | (5.1) | 31 | (13.1) | 32 | (13.6) | 36 | (15.3) | 125 | (53.0) |
| Pregnant Status | No | 5,582 | 218 | (3.9) | 767 | (13.7) | 1,631 | (29.2) | 1,231 | (22.1) | 1,735 | (31.1) |
|  | Yes | 10 | - | - | 3 | (30.0) | 7 | (70.0) | - | - | - | - |
| Chronic Disease | No | 4,227 | 208 | (4.9) | 723 | (17.1) | 1,451 | (34.3) | 908 | (21.5) | 937 | (22.2) |
|  | Yes | 1,365 | 10 | (0.7) | 47 | (3.4) | 187 | (13.7) | 323 | (23.7) | 798 | (58.5) |
