# Supplementary material for: Socioeconomic differences in influenza vaccination coverage by government financial support status: A population-based study in Korea
Source: PLoS One. 2026 Aug 13;21(8):e0353764. doi: 10.1371/journal.pone.0353764 (PMC13472430; doi:10.1371/journal.pone.0353764)
Supplement: Supplementary file 2 [file pone.0353764.s002.docx]

Supporting Information 2. Questionnaire and Variables of Korea National Health and Nutrition Examination Survey

| **Measure** | **KNHANES Questionnaire and Variables** | **Variable Name** |
| --- | --- | --- |
| Sex | What is your gender?  1.Male / 2. Female | Sex |
| Number of Household Member | Number of Household?   1. 1 person 2. 2 people 3. 3 people 4. 4 people 5. 5 people 6. 6 people and above   9. Don’t know | Cfam |
| Residency | Residence Area?   1. Urban (“Dong” Unit) / 2. Rural (“Myeon”, “Eup” Unit) | Town_t |
| Education Level | What is your educational level? (Dropouts are not included in graduation)   1. Elementary school graduate or lower 2. Middle school graduate 3. High school graduate 4. College (University) graduate or higher | Edu |
| Household Income Level | Income quartile (household)? (Refer to the quartile division standard amount)   1. Lowest 2. Middle-Low 3. Middle-High 4. Highest | Ho_incm |
| Occupation Type | What is your job?   1. Managers, Professionals, and related workers 2. Clerks 3. Service workers, Sales workers 4. Skilled agricultural, forestry, and fishery workers 5. Craft and related trades workers, Equipment, machine operating, and assembling workers 6. Elementary workers 7. Unemployed (Housewives, Students) | Occp |
| Economic Activity Status | Have you worked for more than one hour for income or worked as an unpaid family worker for more than 18 hours in the past week? This includes those who were employed but temporarily furloughed.   1. Yes / 2. No / 8. Under 15 years of age | Ec1_1 |
| Type of Health Insurance | Type of Health Insurance  10. Self-employed   20. Employee 30. Medical aid 99. Don’t know or No response | Tins |
| Marital Status | Have you ever been married?   1. Yes / 2. No / 9. Don't know, No response | Marri_1 |
| Chronic Disease | Have you been diagnosed with the disease by a doctor?   - Diabetes mellitus - Heart diseases (coronary heart disease, myocardial infarction, angina, stroke) - Lung diseases (pulmonary tuberculosis, bronchial asthma, chronic bronchitis, emphysema) - Liver diseases (chronic hepatitis, liver cirrhosis) - Kidney diseases (chronic renal disease) - Cancer (Stomach, Liver, Colon, Breast, Cervical, Lung, Other cancer exclude thyroid cancer)   0.No / 1. Yes / 8. Not applicable (Adolescents, Children) / 9. Don't know, No response | de1_dg  Di6_dg, Di5_dg, Di3_dg  dj2_dg, dj4_dg,  dk8_dg, dk9_dg, dk4_dg  Dn1_dg,  dc1_dg, dc2_dg, dc3_dg, dc4_dg, dc5_dg, dc6_dg, dc11_dg |
| Pregnancy | Are you currently pregnant?   1. Yes / 2. No | HE_prg |
| Vaccination | Have you been vaccinated against influenza (seasonal flu) in the past year?   1. Yes / 2. No | Bh9_11 |
