# Supplementary material for: Socioeconomic differences in influenza vaccination coverage by government financial support status: A population-based study in Korea
Source: PLoS One. 2026 Aug 13;21(8):e0353764. doi: 10.1371/journal.pone.0353764 (PMC13472430; doi:10.1371/journal.pone.0353764)
Supplement: Supplementary file 3 [file pone.0353764.s003.docx]

Supporting Information 3. Definition of the Government Financial Support Group for Influenza in the 2021 Korean National Immunization Program

Targets of the National Influenza Vaccination Support Program for the 2020-2021 season, as follows:

1) Age criteria: Children aged 6 months to adolescents under 18 years of age, and the elderly over 62 years of age.

2) Pregnant women

3) Individuals with chronic diseases: Following government recommendations, individuals diagnosed with diabetes, heart disease(angina pectoris, myocardial infarction, and stroke), lung disease(pulmonary tuberculosis and asthma), liver disease(hepatitis B, hepatitis C, and cirrhosis), kidney disease, and cancer(stomach, liver, colon, breast, cervical, lung, and others excluding thyroid) were classified as having chronic diseases.

Targets of the National Influenza Vaccination Support Program for the 2019-2020 season, as follows:

1) Age criteria: Children aged 6 months to 59 months years of age, and the elderly over 65 years of age.

2) Pregnant women

3) Individuals with chronic diseases: Following government recommendations, individuals diagnosed with diabetes, heart disease(angina pectoris, myocardial infarction, and stroke), lung disease(pulmonary tuberculosis and asthma), liver disease(hepatitis B, hepatitis C, and cirrhosis), kidney disease, and cancer(stomach, liver, colon, breast, cervical, lung, and others excluding thyroid) were classified as having chronic diseases.

* Expansion of Eligibility by Age Group

1) To prevent influenza outbreaks caused by communal living: (Existing) Children aged 6 months to 59 months --> (Expanded) Children and adolescents aged 6 months to 18 years.

2) To prevent community influenza outbreaks: (Existing) Seniors aged 65 and older --> (Expanded) Seniors aged 62 and older: Temporary expansion of vaccination for seniors aged 62 to 64.
