# Supplementary material for: Socioeconomic differences in influenza vaccination coverage by government financial support status: A population-based study in Korea
Source: PLoS One. 2026 Aug 13;21(8):e0353764. doi: 10.1371/journal.pone.0353764 (PMC13472430; doi:10.1371/journal.pone.0353764)
Supplement: Supplementary file 4 [file pone.0353764.s004.docx]

Supporting Information 4. The results of model fit statistics

| Variable | *Model* | AIC | *SC* | *-2 Log L* |
| --- | --- | --- | --- | --- |
| Financial support group | 1 | 20695164 | 20695178 | 20695162 |
|  | 2 * | 20528004 | 20528033 | 20528000 |
|  |  |  |  |  |
| Variable | *Model* | AIC | *SC* | *-2 Log L* |
| Non-Financial support group | 1 | 33524457 | 33524472 | 33524455 |
|  | 2 * | 32512199 | 32512395 | 32512173 |
| AIC: Akaike’s Information Criterion, SC: Schwarz Criterion  *: The lower AIC indicates that the adjusted model provided a better fit to the data. | | | | |

Supporting Information 5. Among variables using variance inflation factors (VIFs) in each variable and entire variables

| Variables in each variable | VIFs * |
| --- | --- |
| Education ↔ House Income | 1.19 |
| Education ↔ Economic Activity Status | 1.07 |
| House Income ↔ Economic Activity Status | 1.06 |
|  |  |
| Variables in entire variables | VIFs * |
| Financial Supported | 1.52 |
| Sex | 1.06 |
| Number of Households Member | 1.12 |
| Residency Area | 1.05 |
| Level of education | 1.51 |
| Household income | 1.36 |
| Economic activity status | 1.19 |
| Marital Status | 1.11 |
| Health Insurance Type | 1.02 |
| *: As the VIF values for all variables were significantly lower than the generally accepted threshold (VIF < 5) multicollinearity was found to be not an issue. | |

Supporting Information 6. Effect of financial support on vaccination by socioeconomic status (interaction model)

| Variable | *df* | Estimate * | *SE* | *t* | Pr > \|t\| |
| --- | --- | --- | --- | --- | --- |
| Financial support | 1 | 1.4667 | 0.1675 | 8.76 | <.0001 |
| Education | 1 | 0.1298 | 0.1609 | 0.81 | 0.4209 |
|  | 2 | 0.0352 | 0.1327 | 0.27 | 0.7910 |
| Financial support × Education | 1 | -0.6740 | 0.2038 | -3.31 | 0.0012 |
|  | 2 | -1.2520 | 0.2231 | -5.61 | <.0001 |
|  |  |  |  |  |  |
| Variable | *df* | Estimate * | *SE* | *t* | Pr > \|t\| |
| Financial support | 1 | 1.8813 | 0.2191 | 8.58 | <.0001 |
| House Income | 1 | 0.7979 | 0.2231 | 3.58 | 0.0005 |
|  | 2 | 0.8711 | 0.2200 | 3.96 | 0.0001 |
|  | 3 | 0.9410 | 0.2266 | 4.15 | <.0001 |
| Financial support × House Income | 1 | -0.9567 | 0.2516 | -3.80 | 0.0002 |
|  | 2 | -1.4070 | 0.2368 | -5.94 | <.0001 |
|  | 3 | -1.4915 | 0.2471 | -6.04 | <.0001 |
|  |  |  |  |  |  |
| Variable | *df* | Estimate * | *SE* | *t* | Pr > \|t\| |
| Financial support | 1 | 0.5188 | 0.0987 | 5.26 | <.0001 |
| Economic Activity | 1 | 0.0344 | 0.1017 | 0.34 | 0.7359 |
| Financial support × Economic Activity | 1 | 0.4534 | 0.1335 | 3.40 | 0.0009 |
| *: Interaction estimates | | | | | |
