# Supplementary material for: Socioeconomic differences in influenza vaccination coverage by government financial support status: A population-based study in Korea
Source: PLoS One. 2026 Aug 13;21(8):e0353764. doi: 10.1371/journal.pone.0353764 (PMC13472430; doi:10.1371/journal.pone.0353764)
Supplement: Supplementary file 5 [file pone.0353764.s005.docx]

**Editorial Reviewer Report**

**Manuscript Title:**

Socioeconomic differences in influenza vaccination coverage by government financial support status: A population-based study in Korea

**Manuscript ID:**

PONE-D-26-08153

**Over all comments**

This manuscript investigates the association between government financial support and influenza vaccination coverage using nationally representative data from the Korea National Health and Nutrition Examination Survey (KNHANES 2020). The study applies multivariable logistic regression and subgroup analyses to identify socioeconomic factors associated with vaccination uptake among financially supported and non-supported populations. The topic is relevant and timely for public health policy; however, several important methodological, structural, and reporting issues need to be addressed before the manuscript can be considered for publication.

**Major comments**

1. Age and chronic disease are used to define exposure but are not included in the main Multivariable Logistic Regression. These are strong determinants of vaccination behavior and may confound the observed associations. Recommended to Include age and chronic disease variables in all regression models or justify their exclusion. Consider stratified or sensitivity analyses.
2. The definition of financial support eligibility combines national policy, COVID-19 expansion, and potentially local programs without clear distinction. Provide a clear and structured definition of eligibility criteria (preferably in a table or appendix) for better understanding of the exposure outcome
3. It appears to be a potential issue of residual confounding, as *occupation* is included in the descriptive analysis but excluded from the regression models without justification. Given that occupation is an important determinant of healthcare access and vaccination uptake, its exclusion may bias the results. Please clearly justify the covariate selection strategy and assess potential multicollinearity among socioeconomic variables (e.g., education, income, occupation, and economic activity). Consider including occupation in the regression models or provide a rationale for its exclusion.
4. In Section 2.2 (Study design, lines 127–133), the study design is described by directly referencing result tables (Table 1–4). This approach is methodologically inappropriate. The *study design section should describe the analytical framework and methods*, not refer to specific result tables. Tables belong to the Results section and should not be embedded or cited as part of the study design.
5. The outcome measure should be described more carefully in relation to timing and recall.
   Vaccination status is based on self-report to the question asking whether the participant had been vaccinated against influenza in the past year. Because influenza vaccination is seasonal and the policy window is specific to the 2020–2021 season, the timing linkage between the reported vaccination and the exposure definition should be clarified. Please clarify the survey timing, vaccination reference period, and why this aligns appropriately with the 2020–2021 support policy.
6. In Section 2.6 (Statistical analysis, lines 183–186), the manuscript includes the following sentence: “All participants provided written informed consent… approved by the Institutional Review Board…” This statement pertains to ethical approval and informed consent and is not appropriate within the Statistical Analysis subsection. Therefore, remove the ethical statement from Section 2.6 (Statistical Analysis) and include at the end of the Methods section under a separate subheading
7. Although KNHANES uses a complex sampling design, details on weighting, stratification, and clustering are insufficient. Authors are required to specify Survey weights used, PSU (cluster) and strata variables using SAS procedures (e.g., PROC SURVEYLOGISTIC)
8. The manuscript mentions AIC but does not report values or other diagnostics. Please check actual AIC results, model fit or justify the reason.
9. The Results section (Section 3) is currently presented as a continuous narrative, which makes it difficult to follow and interpret key findings. The absence of subheadings leads to a monotonous flow, despite multiple distinct analyses being conducted (descriptive, regression, subgroup analyses). It may reduce readability and clarity for readers. Please restructure the Results section using clear, logical subheadings that reflect the analytical steps.
10. Separate models are presented for supported and non-supported groups, but no interaction tests are conducted. If possible, analyze the test for interaction effects between financial support eligibility and key SES variables. Logical regression tables: Statistical Significance Not Clearly Indicated, “Bold characteristics” noted but not visually clear. Revise it adding symbols (*, **)
11. In the Discussion section, several comparisons with prior studies are written in the present tense, for example phrases such as “previous studies show” or “research supports.” However, when referring to published studies and completed research, the appropriate tense is typically past tense
12. There are internal inconsistencies between the manuscript file and submission metadata that should be corrected: the submission metadata states that there was “no specific funding,” while the manuscript’s Funding section reports National Cancer Center support under grant NCC-24H1052-3. Likewise, the data availability language says restrictions apply, but then states that all KNHANES files are available from the KDCA database.
13. There are major issues in reference style. Please revise it with appropriate DOI, URL and access date wherever required in the following:

- Ref 1: WHO webpage (missing access date)
- Ref 6: WHO paper (unclear publication format)
- Ref 16: KDCA guideline (incomplete citation -no publisher details)
- Ref 17: OECD (incomplete, missing URL or report details)
- Ref 24: Appears to be unpublished or unclear source

Acronyms issues in the reference:

- Use full institutional names on first mention, then abbreviations consistently:
- World Health Organization (WHO)
- Korea Disease Control and Prevention Agency (KDCA)
