# Supplementary material for: Socioeconomic differences in influenza vaccination coverage by government financial support status: A population-based study in Korea
Source: PLoS One. 2026 Aug 13;21(8):e0353764. doi: 10.1371/journal.pone.0353764 (PMC13472430; doi:10.1371/journal.pone.0353764)
Supplement: Supplementary file 6 [file pone.0353764.s007.docx]

**Editorial Reviewer Report**

**Manuscript Title:**

Socioeconomic differences in influenza vaccination coverage by government financial support status: A population-based study in Korea

**Manuscript ID:**

PONE-D-26-08153

**Over all comments**

This manuscript investigates the association between government financial support and influenza vaccination coverage using nationally representative data from the Korea National Health and Nutrition Examination Survey (KNHANES 2020). The study applies multivariable logistic regression and subgroup analyses to identify socioeconomic factors associated with vaccination uptake among financially supported and non-supported populations. The topic is relevant and timely for public health policy; however, several important methodological, structural, and reporting issues need to be addressed before the manuscript can be considered for publication.

🡪 **RESPONSE:** We sincerely appreciate your considerable comments and recommendations. We have taken your comment seriously and responded to it this time. The modified paragraphs of the text are in red font in the revised manuscript.

**Major comments**

1. Age and chronic disease are used to define exposure but are not included in the main Multivariable Logistic Regression. These are strong determinants of vaccination behavior and may confound the observed associations. Recommended to Include age and chronic disease variables in all regression models or justify their exclusion. Consider stratified or sensitivity analyses.

🡪 **RESPONSE:** We appreciate thoughtful comment.

The variables of age and chronic disease you mentioned are determinants that strongly influence vaccination behavior. When we first designed this study, we proposed age and the presence of chronic disease as covariates. However, during the process of defining the financial support group, we confirmed that government financial support was received based precisely on criteria regarding age and chronic disease. Due to the multicollinearity issue arising from this, we excluded age and chronic disease from the covariates. Therefore, the overall logistic regression analysis was conducted by excluding age and chronic disease. (page 7 line 153 – page 8 line 163)

Definition of Financial Support Group:

1) Age criteria: Children and adolescents under 18 years of age, and the elderly over 62 years of age.

2) Pregnant women: 10 of the study participants were identified as pregnant, and all were considered to have received free government-supported vaccination.

3) Individuals with chronic diseases: Following government recommendations, individuals diagnosed with diabetes, heart disease(angina pectoris, myocardial infarction, and stroke), lung disease(pulmonary tuberculosis and asthma), liver disease(hepatitis B, hepatitis C, and cirrhosis), kidney disease, and cancer(stomach, liver, colon, breast, cervical, lung, and others excluding thyroid) were classified as having chronic diseases.

2. The definition of financial support eligibility combines national policy, COVID-19 expansion, and potentially local programs without clear distinction. Provide a clear and structured definition of eligibility criteria (preferably in a table or appendix) for better understanding of the exposure outcome.

🡪 **RESPONSE:** It is clearly a limitation of our study that the vaccination local programs for each region, as suggested by the reviewer, were not included in the definition for supported group. The Republic of Korea consists of 17 metropolitan and provincial governments and 226 basic local governments. There were limitations in applying the different policies of each of the 243 local governments. Therefore, we adopted only the policies provided by the central government as free vaccination policies. We have added this to the limitations section of the study. (page 15 line 340-342)

Furthermore, we consider the financial support group that who received the central government’s policies, however, we couldn’t reflect the individuals who received the local government’s support.

Additionally, we have clarified the definition of the group that received state financial support during the period of the study data by adding it to the main manuscript (page 7 line 147 – page 8 line 163) and Supplementary3.

However, during the 2020-2021 COVID-19 pandemic, the age criteria were temporarily expanded for the following reasons: 1) To prevent influenza outbreaks caused by communal living, the existing age criterion for children aged 6 months to 59 months was expanded to include children and adolescents aged 6 months to 18 years. 2) To prevent community influenza outbreaks, the existing age criterion for seniors aged 65 and older was expanded to include seniors aged 62 and older.

1) Age criteria: Children and adolescents under 18 years of age, and the elderly over 62 years of age.

2) Pregnant women: 10 of the study participants were identified as pregnant, and all were considered to have received free government-sponsored vaccination.

3) Individuals with chronic diseases: Following government recommendations, individuals diagnosed with diabetes, heart disease(angina pectoris, myocardial infarction, and stroke), lung disease(pulmonary tuberculosis and asthma), liver disease(hepatitis B, hepatitis C, and cirrhosis), kidney disease, and cancer(stomach, liver, colon, breast, cervical, lung, and others excluding thyroid) were classified as having chronic diseases.

3. It appears to be a potential issue of residual confounding, as *occupation* is included in the descriptive analysis but excluded from the regression models without justification. Given that occupation is an important determinant of healthcare access and vaccination uptake, its exclusion may bias the results. Please clearly justify the covariate selection strategy and assess potential multicollinearity among socioeconomic variables (e.g., education, income, occupation, and economic activity). Consider including occupation in the regression models or provide a rationale for its exclusion.

🡪 **RESPONSE:** Thank you for your thorough review. Although occupation is an important determinant of healthcare access, it showed limited utility in our data. The variable included multiple categories, with a substantial proportion of participants classified as unemployed (41.7%), and vaccination rates were relatively homogeneous across groups, indicating limited discriminatory power. Accordingly, occupation was excluded from the final model, and economic activity status was used instead to improve model parsimony and interpretability. We deleted the variables and frequencies regarding occupation types presented in Table 1 to minimize confusion. (page 18)

Based on your feedback, we evaluated multicollinearity using variance inflation factors (VIFs) for each variable (Table 1) and the entire model (Table 2). As the VIF values for all variables were significantly lower than the generally accepted threshold (VIF < 5), multicollinearity was found to be not an issue. A summary of the VIF values is provided below and supplementary4.

Supplementary 4. Among variables using variance inflation factors (VIFs) in each variable and entire variables

| Variables in each variable | VIFs * |
| --- | --- |
| Education ↔ House Income | 1.19 |
| Education ↔ Economic Activity Status | 1.07 |
| House Income ↔ Economic Activity Status | 1.06 |
|  |  |
| Variables in entire variables | VIFs * |
| Financial Supported | 1.52 |
| Sex | 1.06 |
| Number of Households Member | 1.12 |
| Residency Area | 1.05 |
| Level of education | 1.51 |
| Household income | 1.36 |
| Economic activity status | 1.19 |
| Marital Status | 1.11 |
| Health Insurance Type | 1.02 |
| *: As the VIF values for all variables were significantly lower than the generally accepted threshold (VIF < 5) multicollinearity was found to be not an issue. | |

4. In Section 2.2 (Study design, lines 127–133), the study design is described by directly referencing result tables (Table 1–4). This approach is methodologically inappropriate. The *study design section should describe the analytical framework and methods*, not refer to specific result tables. Tables belong to the Results section and should not be embedded or cited as part of the study design.

🡪 **RESPONSE:** We fully agree with your comment. We clearly recognized that the table belongs to the results section, so we revised the text accordingly and removed the reference to the results table. (page 6 line 127-133)

5. The outcome measure should be described more carefully in relation to timing and recall.
   Vaccination status is based on self-report to the question asking whether the participant had been vaccinated against influenza in the past year. Because influenza vaccination is seasonal and the policy window is specific to the 2020–2021 season, the timing linkage between the reported vaccination and the exposure definition should be clarified. Please clarify the survey timing, vaccination reference period, and why this aligns appropriately with the 2020–2021 support policy.

🡪 **RESPONSE:** The point you raised is the most significant limitation of this study. We have revised the part regarding the inability to infer causal relationships regarding policy effects due to the cross-sectional study design explained in the limitations section as follows. I hope this has been sufficiently explained as a limitation. (page 15 line 329-336)

This study also has several limitations. First, since influenza vaccination status was determined through a self-reporting method based on respondents' recalls of vaccinations over the past 12 months, there is a possibility of recall bias. Second, because the survey was conducted over the entire year of 2020 (January to December), the 12-month recall period covers two flu seasons (2019–2020 and 2020–2021) and aligns only partially with the 2020 seasonal influenza vaccination guidelines published in July 2020. This temporal overlap may lead to undifferentiated exposure misclassification. Finally, due to the cross-sectional study design, it is not possible to infer causal relationships regarding policy effects.

6. In Section 2.6 (Statistical analysis, lines 183–186), the manuscript includes the following sentence: “All participants provided written informed consent… approved by the Institutional Review Board…” This statement pertains to ethical approval and informed consent and is not appropriate within the Statistical Analysis subsection. Therefore, remove the ethical statement from Section 2.6 (Statistical Analysis) and include at the end of the Methods section under a separate subheading

**🡪 RESPONSE:** Reflecting your comments, we have separated the ethical statement section from Section 2.6 (statistical analysis) and revised it into a separate Section 2.7. (page 9 line 190-194)

7. Although KNHANES uses a complex sampling design, details on weighting, stratification, and clustering are insufficient. Authors are required to specify Survey weights used, PSU (cluster) and strata variables using SAS procedures (e.g., PROC SURVEYLOGISTIC)

🡪 **RESPONSE:** Thank you for your thorough review.

Based on your comment, we have modified the analysis methods section of the manuscript. (page 8 line 181 – page 9 line 188)

2.6 Statistical analysis

SAS software version 9.4 (SAS Institute, Cary, NC, USA) was used for all data analyses. For categorical variables, the chi-square test was used to compare the proportions of sociodemographic characteristics between vaccinated and unvaccinated individuals. Multivariate logistic regression was used to analyze the impact on vaccination rates after adjusting for covariates (Proc Surveylogistic). Data from the Korea National Health and Nutrition Examination Survey were extracted using a two-stage stratified cluster sample design, and strata(Kstrata), clusters(PSU), and weights(wt_itvex) were considered and reflected in the analysis.

8. The manuscript mentions AIC but does not report values or other diagnostics. Please check actual AIC results, model fit or justify the reason.

🡪 **RESPONSE:** As your feedback, we modified the manuscript clearer, and we presented the results of model fit statistics in supplementary6. (page 11 line 241-244)

Model fit was assessed using Akaike’s Information Criterion (AIC). The intercept-only model had an AIC of [20695164], whereas the fully adjusted model had a lower AIC of [20528004]. The reduction in AIC indicates improved model fit. Therefore, the fully adjusted model (Model 2) was selected for inference (Supplementary6).

Supplementary 6. The results of model fit statistics

| Variable | *Model* | AIC | *SC* | *-2 Log L* |
| --- | --- | --- | --- | --- |
| Financial support group | 1 | 20695164 | 20695178 | 20695162 |
|  | 2 * | 20528004 | 20528033 | 20528000 |
|  |  |  |  |  |
| Variable | *Model* | AIC | *SC* | *-2 Log L* |
| Non-Financial support group | 1 | 33524457 | 33524472 | 33524455 |
|  | 2 * | 32512199 | 32512395 | 32512173 |
| AIC: Akaike’s Information Criterion, SC: Schwarz Criterion  *: The lower AIC indicates that the adjusted model provided a better fit to the data. | | | | |

9. The Results section (Section 3) is currently presented as a continuous narrative, which makes it difficult to follow and interpret key findings. The absence of subheadings leads to a monotonous flow, despite multiple distinct analyses being conducted (descriptive, regression, subgroup analyses). It may reduce readability and clarity for readers. Please restructure the Results section using clear, logical subheadings that reflect the analytical steps.

🡪 **RESPONSE:** To clear the results, we restructured the results section as follows and modified in manuscript. (page 9 line 197 – page 11 line 244)

3.1. Study and participant characteristics

3.2. Factors associated to influenza vaccination

3.3. Difference between the Financially Supported Group and the Non-Funded Group

10. Separate models are presented for supported and non-supported groups, but no interaction tests are conducted. If possible, analyze the test for interaction effects between financial support eligibility and key SES variables.

Logical regression tables: Statistical Significance Not Clearly Indicated, “Bold characteristics” noted but not visually clear. Revise it adding symbols (*, **)

🡪 **RESPONSE:** We appreciate this comment.

We analyzed the interaction effects between financial support and major socioeconomic variables. The major socioeconomic variables included education level, household income level, and economic activity status. The results of the interaction analysis between each variable and financial support showed that the effect of financial support decreased as education level increased, and the effect of financial support decreased as income level increased. However, the results of the interaction analysis with the economic activity variable showed that the effect of financial support increased in the economically active group. Further details are attached in Supplementary5.

Supplementary 5. Effect of financial support on vaccination by socioeconomic status (interaction model)

| Variable | *df* | Estimate * | *SE* | *t* | Pr > \|t\| |
| --- | --- | --- | --- | --- | --- |
| Financial support | 1 | 1.4667 | 0.1675 | 8.76 | <.0001 |
| Education | 1 | 0.1298 | 0.1609 | 0.81 | 0.4209 |
|  | 2 | 0.0352 | 0.1327 | 0.27 | 0.7910 |
| Financial support × Education | 1 | -0.6740 | 0.2038 | -3.31 | 0.0012 |
|  | 2 | -1.2520 | 0.2231 | -5.61 | <.0001 |
|  |  |  |  |  |  |
| Variable | *df* | Estimate * | *SE* | *t* | Pr > \|t\| |
| Financial support | 1 | 1.8813 | 0.2191 | 8.58 | <.0001 |
| House Income | 1 | 0.7979 | 0.2231 | 3.58 | 0.0005 |
|  | 2 | 0.8711 | 0.2200 | 3.96 | 0.0001 |
|  | 3 | 0.9410 | 0.2266 | 4.15 | <.0001 |
| Financial support × House Income | 1 | -0.9567 | 0.2516 | -3.80 | 0.0002 |
|  | 2 | -1.4070 | 0.2368 | -5.94 | <.0001 |
|  | 3 | -1.4915 | 0.2471 | -6.04 | <.0001 |
|  |  |  |  |  |  |
| Variable | *df* | Estimate * | *SE* | *t* | Pr > \|t\| |
| Financial support | 1 | 0.5188 | 0.0987 | 5.26 | <.0001 |
| Economic Activity | 1 | 0.0344 | 0.1017 | 0.34 | 0.7359 |
| Financial support × Economic Activity | 1 | 0.4534 | 0.1335 | 3.40 | 0.0009 |
| *: Interaction estimates | | | | | |

🡪 **RESPONSE:** In the analysis table you mentioned, since the bolded parts were not clearly visible, we replaced them all with asterisks (*) to make the statistical significance clearly visible. (Table 2-4) (page 19-21)

11. In the Discussion section, several comparisons with prior studies are written in the present tense, for example phrases such as “previous studies show” or “research supports.” However, when referring to published studies and completed research, the appropriate tense is typically past tense

🡪 **RESPONSE:** We have edited and corrected the English text throughout the entire manuscript. Thank you for your detailed comments.

12. There are internal inconsistencies between the manuscript file and submission metadata that should be corrected: the submission metadata states that there was “no specific funding,” while the manuscript’s Funding section reports National Cancer Center support under grant NCC-24H1052-3. Likewise, the data availability language says restrictions apply, but then states that all KNHANES files are available from the KDCA database.

**🡪 RESPONSE:** This study is a secondary data analysis using data provided by the Korea Disease Control and Prevention Agency (KDCA). The authors received no specific funding for this work. Publication costs (article processing charges) were covered by institutional support.

13. There are major issues in reference style. Please revise it with appropriate DOI, URL and access date wherever required in the following:

- Ref 1: WHO webpage (missing access date)
- Ref 6: WHO paper (unclear publication format)
- Ref 16: KDCA guideline (incomplete citation -no publisher details)
- Ref 17: OECD (incomplete, missing URL or report details)
- Ref 24: Appears to be unpublished or unclear source

**🡪 RESPONSE:** We sincerely appreciate your detailed feedback. We accessed the webpage anew and modified it by entering the appropriate DOI, URL, and access date for each. (page 22 line 398 – page 24 line 494)

**🡪 Ref 24:** The cited work has been made publicly available in a preprint form by the same authors, as it was submitted to another journal. In line with common editorial practices and to avoid potential issues related to similarity checks during peer review, we have cited the preprint version to ensure transparency and accessibility of the referenced work. Please consider about it.

Acronyms issues in the reference:

- Use full institutional names on first mention, then abbreviations consistently:
- World Health Organization (WHO)
- Korea Disease Control and Prevention Agency (KDCA)

**🡪 RESPONSE:** We have standardized the criteria for presenting abbreviations appearing in the manuscript.
