# Supplementary material for: Socioeconomic differences in influenza vaccination coverage by government financial support status: A population-based study in Korea
Source: PLoS One. 2026 Aug 13;21(8):e0353764. doi: 10.1371/journal.pone.0353764 (PMC13472430; doi:10.1371/journal.pone.0353764)
Supplement: Peer review history

# Peer review: Decision Letter 0

**DOI:** 10.1371/journal.pone.0353764.r001

**Authors:** Vasuki Rajaguru

13 Apr 2026

Dear Dr. Shin,

Thank you for submitting your manuscript to PLOS ONE. After careful consideration, we feel that it has merit but does not fully meet PLOS ONE’s publication criteria as it currently stands. Therefore, we invite you to submit a revised version of the manuscript that addresses the points raised during the review process.

Please submit your revised manuscript by May 28 2026 11:59PM. If you will need more time than this to complete your revisions, please reply to this message or contact the journal office at plosone@plos.org. When you're ready to submit your revision, log on to https://www.editorialmanager.com/pone/ and select the 'Submissions Needing Revision' folder to locate your manuscript file.

- A letter that responds to each point raised by the academic editor and reviewer(s). You should upload this letter as a separate file labeled 'Response to Reviewers'.
- A marked-up copy of your manuscript that highlights changes made to the original version. You should upload this as a separate file labeled 'Revised Manuscript with Track Changes'.
- An unmarked version of your revised paper without tracked changes. You should upload this as a separate file labeled 'Manuscript'.

If you would like to make changes to your financial disclosure, please include your updated statement in your cover letter. Guidelines for resubmitting your figure files are available below the reviewer comments at the end of this letter.

If applicable, we recommend that you deposit your laboratory protocols in protocols.io to enhance the reproducibility of your results. Protocols.io assigns your protocol its own identifier (DOI) so that it can be cited independently in the future. For instructions see: https://journals.plos.org/plosone/s/submission-guidelines#loc-laboratory-protocols. Additionally, PLOS ONE offers an option for publishing peer-reviewed Lab Protocol articles, which describe protocols hosted on protocols.io. Read more information on sharing protocols at https://plos.org/protocols?utm_medium=editorial-email&utm_source=authorletters&utm_campaign=protocols.

As the corresponding author, your ORCID iD is verified in the submission system and will appear in the published article. PLOS supports the use of ORCID, and we encourage all coauthors to register for an ORCID iD and use it as well. Please encourage your coauthors to verify their ORCID iD within the submission system before final acceptance, as unverified ORCID iDs will not appear in the published article. *Only*  the individual author can complete the verification step; PLOS staff *cannot*  verify ORCID iDs on behalf of authors.

We look forward to receiving your revised manuscript.

Kind regards,

Vasuki Rajaguru, PhD

Academic Editor

PLOS One

Journal Requirements:

When submitting your revision, we need you to address these additional requirements.

1. Please ensure that your manuscript meets PLOS ONE's style requirements, including those for file naming. The PLOS ONE style templates can be found at

https://journals.plos.org/plosone/s/file?id=wjVg/PLOSOne_formatting_sample_main_body.pdf and https://journals.plos.org/plosone/s/file?id=ba62/PLOSOne_formatting_sample_title_authors_affiliations.pdf

2. Thank you for stating the following financial disclosure:

[The author(s) received no specific funding for this work.].

At this time, please address the following queries:

a) Please clarify the sources of funding (financial or material support) for your study. List the grants or organizations that supported your study, including funding received from your institution.

b) State what role the funders took in the study. If the funders had no role in your study, please state: “The funders had no role in study design, data collection and analysis, decision to publish, or preparation of the manuscript.”

c) If any authors received a salary from any of your funders, please state which authors and which funders.

d) If you did not receive any funding for this study, please state: “The authors received no specific funding for this work.”

3. Thank you for stating the following in your manuscript:

[This work was supported by the National Cancer Center grant funded by the Korean government, the Republic of Korea under Grant [NCC-24H1052-3].]

We note that you have provided funding information that is not currently declared in your Funding Statement. However, funding information should not appear in the Acknowledgments section or other areas of your manuscript. We will only publish funding information present in the Funding Statement section of the online submission form.

Please remove any funding-related text from the manuscript and let us know how you would like to update your Funding Statement. Currently, your Funding Statement reads as follows:

[The author(s) received no specific funding for this work.]

Please include your amended statements within your cover letter; we will change the online submission form on your behalf.

4. We note that you have indicated that there are restrictions to data sharing for this study. For studies involving human research participant data or other sensitive data, we encourage authors to share de-identified or anonymized data. However, when data cannot be publicly shared for ethical reasons, we allow authors to make their data sets available upon request. For information on unacceptable data access restrictions, please see http://journals.plos.org/plosone/s/data-availability#loc-unacceptable-data-access-restrictions.

Before we proceed with your manuscript, please address the following prompts:

a) If there are ethical or legal restrictions on sharing a de-identified data set, please explain them in detail (e.g., data contain potentially identifying or sensitive patient information, data are owned by a third-party organization, etc.) and who has imposed them (e.g., a Research Ethics Committee or Institutional Review Board, etc.). Please also provide contact information for a data access committee, ethics committee, or other institutional body to which data requests may be sent.

b) If there are no restrictions, please upload the minimal anonymized data set necessary to replicate your study findings to a stable, public repository and provide us with the relevant URLs, DOIs, or accession numbers. Please see http://www.bmj.com/content/340/bmj.c181.long for guidelines on how to de-identify and prepare clinical data for publication. For a list of recommended repositories, please see https://journals.plos.org/plosone/s/recommended-repositories. You also have the option of uploading the data as Supporting Information files, but we would recommend depositing data directly to a data repository if possible.

Please update your Data Availability statement in the submission form accordingly.

5. Please note that your Data Availability Statement is currently missing the repository name and/or the DOI/accession number of each dataset. If your manuscript is accepted for publication, you will be asked to provide these details on a very short timeline. We therefore suggest that you provide this information now, though we will not hold up the peer review process if you are unable.

6. When completing the data availability statement of the submission form, you indicated that you will make your data available on acceptance. We strongly recommend all authors decide on a data sharing plan before acceptance, as the process can be lengthy and hold up publication timelines. Please note that, though access restrictions are acceptable now, your entire data will need to be made freely accessible if your manuscript is accepted for publication. This policy applies to all data except where public deposition would breach compliance with the protocol approved by your research ethics board. If you are unable to adhere to our open data policy, please kindly revise your statement to explain your reasoning and we will seek the editor's input on an exemption. Please be assured that, once you have provided your new statement, the assessment of your exemption will not hold up the peer review process.

7. Your ethics statement should only appear in the Methods section of your manuscript. If your ethics statement is written in any section besides the Methods, please delete it from any other section.

8. Please include a separate caption for each figure in your manuscript.

9. Please ensure that you refer to Figure 1 in your text as, if accepted, production will need this reference to link the reader to the figure.

10. Please upload a copy of “Supplementary 3”, to which you refer in your text on page 10. If the figure is no longer to be included as part of the submission please remove all reference to it within the text.

11. Please include captions for your Supporting Information files at the end of your manuscript, and update any in-text citations to match accordingly. Please see our Supporting Information guidelines for more information: http://journals.plos.org/plosone/s/supporting-information.

If the reviewer comments include a recommendation to cite specific previously published works, please review and evaluate these publications to determine whether they are relevant and should be cited. There is no requirement to cite these works unless the editor has indicated otherwise.

Additional Editor Comments:

This manuscript examines the association between government financial support and influenza vaccination coverage using nationally representative KNHANES 2020 data. The authors conduct multivariable logistic regression and subgroup analyses to assess socioeconomic determinants of vaccination among financially supported and non-supported populations. The topic is relevant to public health policy; however, several important methodological and interpretational issues require substantial revision

[Note: HTML markup is below. Please do not edit.]

Reviewers' comments:

Reviewer's Responses to Questions

**Comments to the Author**

1. Is the manuscript technically sound, and do the data support the conclusions?

Reviewer #1: Yes

Reviewer #2: Yes

Reviewer #3: Partly

**********

2. Has the statistical analysis been performed appropriately and rigorously?

Reviewer #1: Yes

Reviewer #2: Yes

Reviewer #3: Yes

**********

3. Have the authors made all data underlying the findings in their manuscript fully available?

The PLOS Data policy

Reviewer #1: Yes

Reviewer #2: Yes

Reviewer #3: Yes

**********

4. Is the manuscript presented in an intelligible fashion and written in standard English?

Reviewer #1: Yes

Reviewer #2: Yes

Reviewer #3: Yes

**********

Reviewer #1: To improve the manuscript, I recommend that you explicitly address the "blind spots" identified in your analysis within the Discussion and Policy Recommendations sections. Specifically, while the National Immunization Program (NIP) effectively bridges the gap for the elderly and children, your data shows that low-income adults who fall outside these eligibility criteria remain highly vulnerable.

I suggest proposing a "tiered support model" or "income-based vouchers" for those in the lowest income quartiles who are currently in the non-supported group. Furthermore, since your study uses 2020 data, a brief discussion on how the COVID-19 pandemic may have uniquely influenced the vaccination behavior of these "self-paid" individuals would provide essential context. Finally, confirming whether KNHANES sampling weights were applied will strengthen the statistical validity of your conclusions for a national audience. These additions will transform your findings into a more actionable framework for achieving true health equity.

Reviewer #2: The topic of submitted paper, "Socioeconomic differences in influenza vaccination coverage by government financial support status: A population-based study in Korea," effectively explains Korea's national policy, and the section describing the differences in influenza vaccination coverage based on socioeconomic status is a crucial element for policy development. I believe that providing financial support to high-risk and socioeconomically disadvantaged groups is effective for influenza vaccination coverage. Therefore, I consider the results of this paper to be meaningful and should be published, as they can serve as a good case study for other countries.

Reviewer #3: The definition of the financial support group requires clarification. The manuscript appears to classify individuals based on eligibility criteria such as age, pregnancy, and chronic disease status, rather than confirmed receipt of free government-sponsored vaccination. This distinction should be clearly stated. In addition, it is unclear whether all individuals with chronic diseases were eligible for free vaccination under the 2020–2021 national program, as some groups may be recommended for vaccination but not necessarily covered by financial support. Furthermore, depending on the type of vaccine selected, individuals may have received vaccination through paid options even if they were eligible for free vaccination. These issues raise concerns about potential misclassification of the exposure.

The Abstract describes the adjusted odds ratio as a difference in vaccination coverage, which may be misleading and should instead be expressed in terms of odds.

The Introduction is somewhat lengthy and would benefit from being streamlined to focus more clearly on the rationale and study objective.

In the Methods section, descriptions of statistical analyses are included within the Study design subsection. For clarity, it would be preferable to separate the conceptual study design from the statistical analysis and present the analytical methods in a dedicated Statistical analysis section.

The interpretation of the findings in the Discussion appears stronger than can be supported by the study design and exposure definition. The financial support group is defined based on eligibility criteria for the national immunization program, which largely includes specific high-risk populations such as children and older adults, whereas the non-support group represents a substantially different population with distinct baseline characteristics. Therefore, the comparison between these groups may reflect differences in underlying population structure rather than the effect of financial support itself.

As the exposure is based on eligibility rather than actual receipt of free vaccination, the observed differences in vaccination coverage may be driven by these underlying characteristics. Accordingly, interpreting the findings as evidence of the effectiveness of government financial support, as well as the policy implications presented, may go beyond what can be supported by the data. Given the cross-sectional design and the potential for residual confounding and exposure misclassification, a more cautious interpretation is warranted.

These methodological considerations should be more explicitly addressed in the limitations section, particularly with respect to the distinction between eligibility and actual uptake, differences in population structure between comparison groups, and the constraints in drawing policy-relevant conclusions.

**********

what does this mean?). If published, this will include your full peer review and any attached files.

If you choose “no”, your identity will remain anonymous but your review may still be made public.

**Do you want your identity to be public for this peer review?**  For information about this choice, including consent withdrawal, please see our Privacy Policy

Reviewer #1: **Yes:** Yahya Abodea

Reviewer #2: No

Reviewer #3: No

**********

[NOTE: If reviewer comments were submitted as an attachment file, they will be attached to this email and accessible via the submission site. Please log into your account, locate the manuscript record, and check for the action link "View Attachments". If this link does not appear, there are no attachment files.]

To ensure your figures meet our technical requirements, please review our figure guidelines: https://journals.plos.org/plosone/s/figures

You may also use PLOS’s free figure tool, NAAS, to help you prepare publication quality figures: https://journals.plos.org/plosone/s/figures#loc-tools-for-figure-preparation.

NAAS will assess whether your figures meet our technical requirements by comparing each figure against our figure specifications.

Attachment Submitted filename: Major revision_001.docx

# Author comment: Author response to Decision Letter 1

**DOI:** 10.1371/journal.pone.0353764.r002

25 Jun 2026

We have updated to the revision file (the file named 'Revised'—cover letter, manuscript, figure, supplementary).

Attachment Submitted filename: 01.ResponseLetter_Vaccine_20260505.docx

# Editor report: Decision Letter 1

**DOI:** 10.1371/journal.pone.0353764.r003

**Authors:** Vasuki Rajaguru

29 Jun 2026

Socioeconomic differences in influenza vaccination coverage by government financial support status: A population-based study in Korea

PONE-D-26-08153R1

Dear Dr. Kim,

We’re pleased to inform you that your manuscript has been judged scientifically suitable for publication and will be formally accepted for publication once it meets all outstanding technical requirements.

Within one week, you’ll receive an e-mail detailing the required amendments. When these have been addressed, you’ll receive a formal acceptance letter and your manuscript will be scheduled for publication.

An invoice will be generated when your article is formally accepted. Please note, if your institution has a publishing partnership with PLOS and your article meets the relevant criteria, all or part of your publication costs will be covered. Please make sure your user information is up-to-date by logging into Editorial Manager at Editorial Manager® and clicking the ‘Update My Information' link at the top of the page. For questions related to billing, please contact billing support.

Congratulations and thank you for submitting your manuscript to PLOS One.

Kind regards,

Vasuki Rajaguru, PhD

Academic Editor

PLOS One

# Editor report: Acceptance letter

**DOI:** 10.1371/journal.pone.0353764.r004

**Authors:** Vasuki Rajaguru

PONE-D-26-08153R1

PLOS One

Dear Dr. KIM,

I'm pleased to inform you that your manuscript has been deemed suitable for publication in PLOS One. Congratulations! Your manuscript is now being handed over to our production team.

At this stage, our production department will prepare your paper for publication. This includes ensuring the following:

* All references, tables, and figures are properly cited

* All relevant supporting information is included in the manuscript submission,

* There are no issues that prevent the paper from being properly typeset

You will receive further instructions from the production team, including instructions on how to review your proof when it is ready. Please keep in mind that we are working through a large volume of accepted articles, so please give us a few days to review your paper and let you know the next and final steps.

Lastly, if your institution or institutions have a press office, please let them know about your upcoming paper now to help maximize its impact. If they'll be preparing press materials, please inform our press team within the next 48 hours. Your manuscript will remain under strict press embargo until 2 pm Eastern Time on the date of publication. For more information, please contact onepress@plos.org.

You will receive an invoice from PLOS for your publication fee after your manuscript has reached the completed accept phase. If you receive an email requesting payment before acceptance or for any other service, this may be a phishing scheme. Learn how to identify phishing emails and protect your accounts at https://explore.plos.org/phishing.

If we can help with anything else, please email us at customercare@plos.org.

Thank you for submitting your work to PLOS ONE and supporting open access.

Kind regards,

PLOS ONE Editorial Office Staff

on behalf of

Dr. Vasuki Rajaguru

Academic Editor

PLOS One
